# Quantitative microvascular analysis of retinal venous occlusions by spectral domain optical coherence tomography angiography

**DOI:** 10.1371/journal.pone.0176404

**Published:** 2017-04-24

**Authors:** Nicole Koulisis, Alice Y. Kim, Zhongdi Chu, Anoush Shahidzadeh, Bruce Burkemper, Lisa C. Olmos de Koo, Andrew A. Moshfeghi, Hossein Ameri, Carmen A. Puliafito, Veronica L. Isozaki, Ruikang K. Wang, Amir H. Kashani

**Affiliations:** 1 USC Roski Eye Institute, Department of Ophthalmology, Keck School of Medicine, University of Southern California, Los Angeles, California; 2 University of Massachusetts Medical School, Worcester, Massachusetts; 3 Department of Bioengineering, University of Washington, Seattle, Washington; 4 Department of Ophthalmology, University of Washington School of Medicine, Seattle, Washington; Massachusetts Eye & Ear Infirmary, Harvard Medical School, UNITED STATES

## Abstract

**Purpose:**

To quantitatively evaluate the retinal microvasculature in human subjects with retinal venous occlusions (RVO) using optical coherence tomography angiography (OCTA).

**Design:**

Retrospective, cross-sectional, observational case series.

**Participants:**

Sixty subjects (84 eyes) were included (20 BRVO, 14 CRVO, 24 unaffected fellow eyes, and 26 controls).

**Methods:**

OCTA was performed on a prototype, spectral domain-OCTA system in the 3x3mm central macular region. Custom software was used to quantify morphology and density of retinal capillaries using four quantitative parameters. The vasculature of the segmented retinal layers and nonsegmented whole retina were analyzed.

**Main outcome measures:**

Fractal dimension (FD), vessel density (VD), skeletal density (SD), and vessel diameter index (VDI) within the segmented retinal layers and nonsegmented whole retina vasculature.

**Results:**

Nonsegmented analysis of RVO eyes demonstrated significantly lower FD (1.64±0.01 vs 1.715±0.002; p<0.001), VD (0.32±0.01 vs 0.432±0.002; p<0.001), and SD (0.073±0.004 vs 0.099±0.001; p<0.001) compared to controls. Compared to the fellow eye, FD, VD and SD were lower (p<0.001), and VDI was higher (p<0.001). FD, VD, and SD progressively decreased as the extent (or type) of RVO increased (control vs BRVO vs CRVO; p<0.001). In the unaffected fellow eye FD, VD and SD showed significant differences when compared to control eyes or affected RVO eyes (p<0.001).

**Conclusions:**

Quantitative OCTA of the central 3x3mm macular region demonstrates significant differences in capillary density and morphology among subjects with BRVO and CRVO compared to controls or unaffected fellow eyes in all vascular layers. The unaffected fellow eyes also demonstrate significant differences when compared to controls. OCTA allows for noninvasive, layer-specific, quantitative evaluation of RVO-associated microvascular changes.

## Introduction

Retinal venous occlusion (RVO) is the second most common retinal vascular disorder after diabetic retinopathy.[1] An estimated 16.4 million adults worldwide are affected with a prevalence of 4 and 0.8 per 1000 individuals for branch (BRVO) and central retinal vein occlusion (CRVO), respectively.[2] Traditionally, intravenous fluorescein angiography (IVFA) has been used to assess impairments in capillary perfusion (“capillary nonperfusion”). IVFA requires intravenous injection of fluorescein dye, is time-intensive, and carries a risk of serious adverse reactions including anaphylaxis and death.[3]

Optical coherence tomography angiography (OCTA) is a noninvasive tool that allows rapid imaging of retinal capillaries.[4–6] In the 3x3mm foveal region of RVO subjects, OCTA demonstrates impaired vascular perfusion, increased vessel caliber, vessel shunting, intraretinal edema, and enlargement of the foveal avascular zone.[4, 7–11] In conjunction with spectral domain optical coherence tomography (SD-OCT) cross-sectional analysis of macular anatomy, analysis of *en face* OCTA may be as effective as IVFA for evaluating and managing the macular complications of RVO.[4, 12] Furthermore, OCTA conveys previously unattainable, layer-specific information.

The ability to noninvasively and quantitatively evaluate the retinal microvasculature on a regular basis may provide clinicians with an objective method for monitoring RVO disease severity, progression, and response to interventions such as treatment with intravitreal therapies. Recently, a custom algorithm was developed for quantitative evaluation of the retinal capillaries.[13–15] The algorithm assesses vessel density and morphology within OCTA images in a layer-specific manner. Herein, we adapt this algorithm for quantitative analysis of retinal capillaries in RVO and report novel, quantitative changes that may represent promising, noninvasive biomarkers of disease severity.

## Methods

This was a retrospective study of adult, human subjects who underwent standard-of-care clinical evaluation and treatment for RVO. Although this was a retrospective analysis, all subjects including control subjects had provided written informed consent to have their eyes evaluated with an investigational imaging device. Research was conducted in compliance with the Declaration of Helsinki and with approval from the University of Southern California (USC) Institutional Review Board. As part of their routine clinical care, all subjects underwent ophthalmic examination including best-corrected Snellen visual acuity, intraocular pressure, slit lamp examination, indirect ophthalmoscopy, and SD-OCT (Cirrus, Carl Zeiss Meditec, Inc., Dublin, CA; Spectralis, Heidelberg Engineering Inc., Heidelberg, Germany). Fundus photography, wide-field photography (Optos 200Tx, Optos, Dunfermline, Scotland; Spectralis), and IVFA (Optos 200Tx; Spectralis) were performed when indicated, as part of routine care.

Subjects were categorized based on the type of RVO (BRVO vs CRVO) and eyes were categorized by RVO status. RVO status refers to the relationship of the eye of interest to the RVO eye, and was defined as one of the following: RVO eye, unaffected fellow eye, or control eye. The presence of macular edema was determined using clinical examination and SD-OCT by the treating physician. Individuals with significant comorbidities including severe non-proliferative or proliferative diabetic retinopathy, neovascular age-related macular degeneration, retinal arterial occlusion, or previous vitreoretinal surgery were excluded.

Every attempt was made to collect control subjects without any history of vascular disease. However, due to the prevalence of common systemic vascular diseases (e.g. hypertension) in the age-matched population, it was not possible to collect such a cohort in a study of this size. Therefore, subjects with well-controlled diabetes, hypertension and/or hyperlipidemia, and no significant retinal findings were included in the control group. Information on race, which was voluntary and self-designated, was collected to best match RVO subjects with controls.

All subjects underwent imaging at the USC Roski Eye Institute or the Los Angeles County+USC Medical Center from January 2015 through February 2016. OCTA images were acquired on a prototype SD-OCTA system (Carl Zeiss Meditec, Inc., Dublin, CA), with central wavelength of 840 nm and scan speed of 68,000 A-scans per second. The device had standard Cirrus SD-OCT hardware as well as custom acquisition and analysis software supplied by Carl Zeiss Meditec.[6, 13] All subjects were instructed to focus on a fixation target while a 3x3mm, fovea-centered macula scan (300 A-scans per line, 350 B-scans) was obtained. The imaging procedure and scan protocol are detailed elsewhere.[4–6, 14, 15]

**Fig 1:**
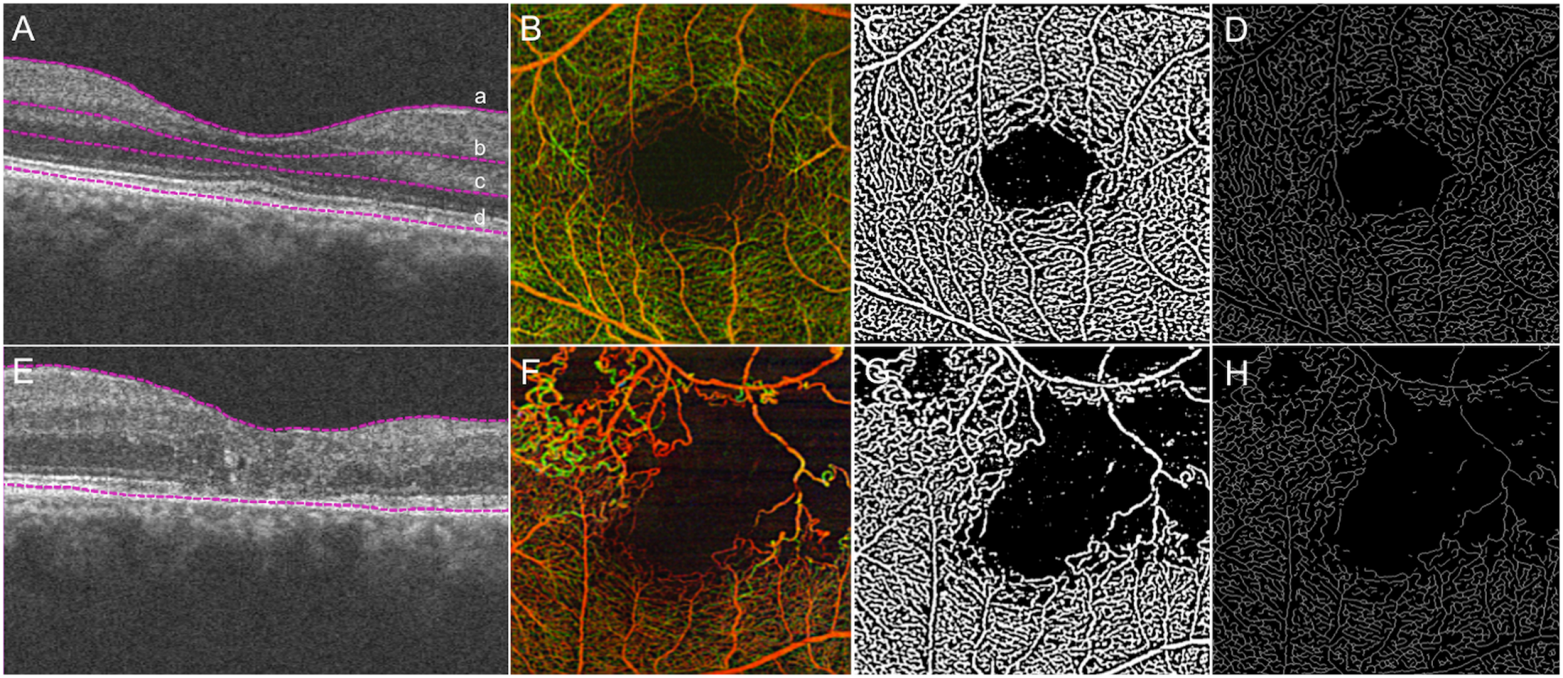
Representative OCTA images of fellow healthy eye and BRVO eye illustrating qualitative changes in capillary density and morphology. (A) B-scan with purple dotted lines delineating the retinal vascular layers of interest in the healthy fellow eye of an RVO subject: Nonsegmented whole retina, NS-RL (a-d); Superficial retina layer, SRL (a-b); Deeper retina layer, DRL (b-c). Corresponding pseudocolored, depth-encoded OCTA map (B), binarized image (C), and skeletonized image (D) of the unaffected eye. (E-H) Corresponding images of the RVO eye, NS-RL analysis.

Automated segmentation was used to delineate the boundaries of three depth-resolved retinal slabs, referred to as the nonsegmented retina (NS-RL), superficial retina layer (SRL), and deep retina layer (DRL).[6] The NS-RL extended from the internal limiting membrane (ILM) to the retinal pigment epithelium (Fig 1Aa–1Ad), the SRL extended from the ILM to the superficial inner plexiform layer (IPL; **a-b**), and the DRL extended from the deep IPL to the superficial outer nuclear layer (**b-c**; see S1 Fig for representative angiograms; available at www.aaojournal.org). It should be noted that the boundaries of these layers are not well defined within the foveal pit where the deep and superficial vasculature merge into a single capillary ring surrounding the fovea. Therefore, the boundaries of the retinal layers within the foveal pit were consistently drawn to include the foveal capillary ring within the superficial retinal layer. This segmentation method was previously shown to have good correlation with histological measurements of retinal capillary density from the literature.[6]

All OCTA results including automated segmentation, B-scans and structural en face images were examined, and images with artifacts were excluded. Manual segmentation was performed in 3 cases where automated segmentation was not possible due to extensive fovea-involving edema. In 2 such cases, segmented analysis was excluded due to distortion of retinal architecture. In cases where distortion of retinal architecture prevented accurate segmentation or assessment of layer-specific perfusion, only NS-RL analysis was performed.

Quantitative analysis with custom MATLAB (R2013b, MathWorks, Inc., Natick, MA) software was used to transform both segmented and nonsegmented angiograms into binarized and skeletonized images (Fig 1), from which four parameters including fractal dimension (FD), vessel density (VD), skeletal density (SD), and vessel diameter index (VDI) were calculated as previously described.[13–15] These parameters are designed to quantitatively characterize capillary density (VD, SD) and capillary morphology (FD, VDI) since both density (“dropout”) and morphology (e.g. vessel dilation) are known variables in the development and progression of retinal vascular disease. VD is derived from the binarized OCTA image and quantifies the percentage of the angiogram area with detectable perfusion. The binarized image can be reduced to a skeletonized representation of retinal vessels by reducing the width of each vessel segment to one pixel. SD is then derived from this skeletonized image and represents the absolute linear distance (length) of blood vessels in the image. Therefore, SD is representative of the length of the entire retinal vascular network independent of vessel caliber. FD quantifies the extent of vessel branching (vessel complexity), and is derived from the skeletonized image using a box-counting method previously described.[13–15] VDI is derived from both binarized and skeletonized images and quantifies the average vascular caliber (vessel diameter). These parameters quantitatively characterize vessel morphology (FD, VDI) and capillary density (SD, VD), and have previously been demonstrated to have very good repeatability and reliability.[13–15]

Eyes with RVO were compared to those from age- and gender-matched controls, and were also compared to the unaffected fellow eye. For the latter analysis, subjects with bilateral RVO or other eye pathology were excluded. All RVO subjects were analyzed collectively and then sub-grouped based on RVO type (CRVO, BRVO) and presence of macular edema.

Statistical analysis was performed using SAS 9.4 (SAS Institute, Inc., Cary, NC). Mean and standard deviation values were reported. Visual acuity was converted to the logarithm of the minimal angle of resolution using the Holladay protocol.[16] Multivariable linear regressions were performed and the generalized estimating equation (GEE) approach was used to control for age, gender, and between-eye correlation when appropriate. Wilcoxon Rank-Sum test was not used because it would not allow controlling for age and gender and between eye correlation. Regression coefficients (β’s), indicating magnitude and direction of between-group differences, were obtained. Model fit was assessed using Pan's quasi-likelihood under the independence model criterion (QIC) statistic (2001) for GEE models, which is a modification of Akaike’s information criterion (AIC), in which the likelihood estimate is replaced by a quasi-likelihood estimate, with an adjustment made for the penalty term. The model fit was sufficient to determine if there was a significant between-group difference in the outcome measure after controlling for potential confounding factors and between-eye correlation. Due to the small sample sizes of the comparison groups, additional nonparametric analyses were also performed to obtain more conservative p-values. The Jonckheere-Terpstra test was used to assess for a trend in OCTA parameters across different RVO types (control vs BRVO vs CRVO eyes), and RVO statuses (control vs fellow unaffected eyes vs RVO eyes). All statistical analyses were considered significant for (α) set to 0.05.

## Results

Sixty subjects (84 eyes; 34 RVO, 24 unaffected fellow eyes, 26 control eyes) were analyzed. The cohort consisted of 20 (58.8%) females with RVO and demonstrated a broad spectrum of disease (14 CRVO, 20 BRVO; Table 1). Twenty-two subjects with RVO (64.7%; 9 CRVO, 13 BRVO) had macular edema on the date of imaging. Twenty (58.8%; 11 CRVO, 9 BRVO) had any history of intravitreal anti-VEGF therapy. Seven subjects (20.6%; 5 CRVO, 2 BRVO) had received anti-VEGF treatment within 60 days of imaging. Two subjects (5.9%; 1 CRVO, 1 BRVO) had history of intravitreal steroid therapy within 180 days of imaging.

**Table 1:** Characteristics of patients with retinal venous occlusion and control subjects.

	Controls N = 26	All RVO N = 34	Subgroups	
			BRVO N = 20	CRVO N = 14
Age, years				
Mean ± SD	60.9 ± 9.6	64.8 ± 8.8	63.3 ± 9.2	67.0 ± 8.1
Range	46–82	46–80	46–80	47–77
Gender				
Male (%)	11 (42.3)	14 (41.2)	8 (40.0)	6 (42.9)
Female	15 (57.7)	20 (58.8)	12 (60.0)	8 (57.1)
Race				
Hispanic (%)	12 (46.2)	14 (41.2)	9 (45.0)	5 (35.7)
Caucasian	6 (23.1)	12 (35.3)	6 (30.0)	6 (42.9)
Asian	6 (23.1)	7 (20.6)	5 (25.0)	2 (14.3)
African American	2 (7.7)	1 (2.9)	0 (0.00)	1 (7.14)
Comorbidities				
Hypertension (%)	13 (50.0)	24 (70.6)	13 (65.0)	11 (78.6)
Hyperlipidemia	8 (30.8)	16 (47.1)	13 (65.0)	3 (21.4)
Diabetes	4 (15.4)	8 (23.5)	3 (15.0)	5 (35.7)
Duration of RVO				
Greater than 1 year (%)	-	20 (58.8)	11 (55.0)	9 (64.3)
2–12 months	-	9 (26.5)	5 (25.0)	4 (28.6)
Less than 2 months	-	5 (14.7)	4 (20.0)	1 (7.14)
Prior Treatments				
Treatment Naïve (%)	-	13 (38.2)	11 (55.0)	2 (14.3)
Anti-VEGF	-	20 (58.8)	9 (45.0)	11 (78.6)
Focal/grid laser	-	5 (14.7)	1 (5.0)	4 (28.6)
Intravitreal steroid	-	3 (8.8)	2 (10.0)	1 (7.1)
Unknown	-	1 (2.9)	0 (0.0)	1 (7.1)
Macular Edema				
Present on imaging date (%)	-	22 (64.7)	13 (65.0)	9 (64.3)
Absent on imaging date	-	12 (35.3)	7 (35.0)	5 (35.7)
BCVA on image date				
logMAR, mean ± SE	-	0.63 ± 0.12	0.31 ± 0.09	1.09 ± 0.23
Snellen equivalent in ft, mean	-	20/86	20/41	20/245
No. eyes, NS-RL analysis				
Compared to controls (%)	-	34 (100)	20 (100)	14 (100)
Compared to other eye	-	24 (70.6)	15 (75.0)	9 (64.3)
No. eyes, Segmented analysis				
Compared to controls (%)	-	32 (94.1)	19 (95.0)	13 (92.9)
Compared to other eye	-	22 (64.7)	14 (70.0)	8 (40.0)

RVO = retinal venous occlusion; BRVO = branch retinal venous occlusion; CRVO = central retinal venous occlusion; SD = standard deviation; BCVA = best-corrected visual acuity; logMAR = logarithm of minimal angle of resolution; SD = standard deviation; anti-VEGF = intravitreal vascular endothelial growth factor inhibitor; ft = feet; No. = number of; NS-RL = nonsegmented retina.

All 34 subjects with RVO underwent NS-RL analysis. Of these, 24 (70.6%) had an unaffected fellow eye that qualified for NS-RL analysis and comparison. Thirty-two RVO eyes (94.1%) qualified for segmented (SRL, DRL) analysis, and of these, 22 (64.7%) had a healthy fellow eye that qualified for segmented analysis.

### Nonsegmented analysis of retinal capillaries for all RVO

**Fig 2:**
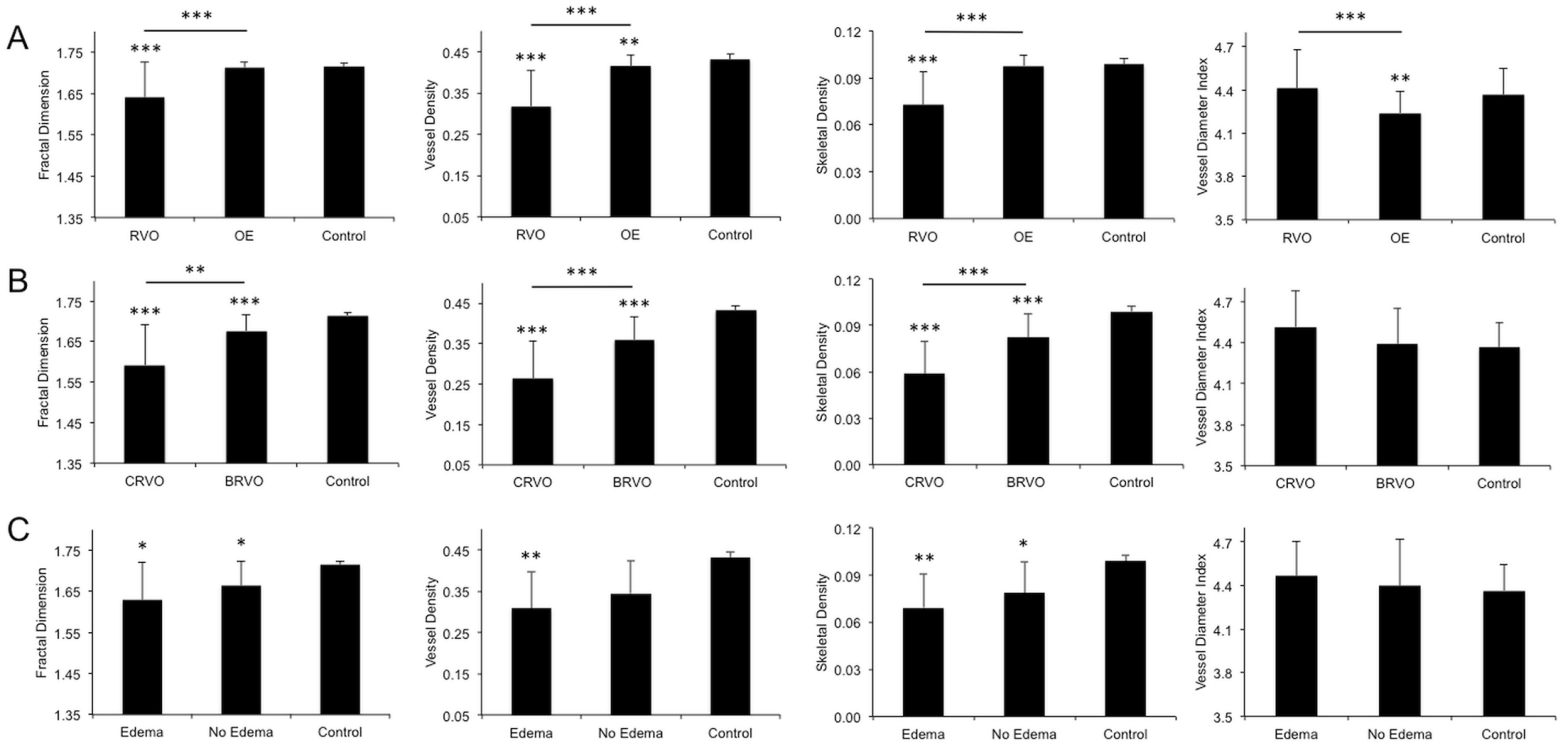
Comparison of quantitative OCTA parameters between RVO eyes and control eyes for nonsegmented layer analysis. Row (A) bar charts illustrate the mean±SD of the quantitative OCTA parameters (Fractal Dimension, Vessel Density, Skeletal Density and Vessel Diameter Index) for eyes with retinal venous occlusion (RVO), the unaffected fellow eye (OE), and control eyes. Row (B) illustrates the same quantitative parameters for central (CRVO) and branch RVO (BRVO) compared to controls. Row (C) illustrates similarly for RVO eyes with and without macular edema. Multivariable linear regressions were performed and the GEE approach was used to control for age, gender, and between-eye correlation when appropriate. The asterisk(s) above the horizontal line indicates that the specified groups were statistically different. All other asterisks compare specified groups to controls. One asterisk, p<0.05; two asterisks, p<0.01; three asterisks, p<0.001.

RVO eyes demonstrated lower mean FD, VD, and SD in the NS-RL compared to controls (p<0.001). VDI was not significantly different (p = 0.06). When RVO eyes were compared to the unaffected fellow eye, mean FD, VD and SD were similarly lower, and VDI significantly greater (p<0.001; Fig 2A). See S1 Table for detailed quantitative comparison. Fig 1 shows images of the NS-RL analysis from a representative subject with RVO.

Sub-group analysis of CRVO and BRVO eyes independently demonstrated lower mean FD, VD, and SD in the NS-RL compared to controls (p<0.001; Fig 2B). The greatest differences were apparent in CRVO subjects (CRVO vs BRVO vs control): FD (1.59±0.10 vs 1.68±0.04 vs 1.72±0.01), VD (0.26±0.09 vs 0.36±0.06 vs 0.43±0.01), SD (0.06±0.02 vs 0.08±0.02 vs 0.099±0.004). Mean VDI was greater in CRVO eyes than either BRVO or control group but without statistical significance (p = 0.07). See Table 2 and S2 Table for detailed quantitative comparison of CRVO, BRVO and control eyes.

**Table 2:** Comparison of quantitative OCTA parameters of BRVO and CRVO eyes compared to controls.

		Controls Mean ± SD	BRVO Mean ± SD	CRVO Mean ± SD	BRVO vs Controls β (95% CI)	p-value*	CRVO vs Controls β (95% CI)	p-value
NS-RL	FD	1.72 ± 0.01	1.68 ± 0.04	1.59 ± 0.10	-0.039 (-0.056, -0.022)	< 0.001	-0.061 (-0.087, -0.034)	< 0.001
	VD	0.43 ± 0.01	0.36 ± 0.06	0.26 ± 0.09	-0.073 (-0.097, -0.048)	< 0.001	-0.082 (-0.016, -0.058)	< 0.001
	SD	0.099 ± 0.004	0.08 ± 0.02	0.06 ± 0.02	-0.017 (-0.023, -0.010)	< 0.001	-0.020 (-0.025, -0.014)	< 0.001
	VDI	4.37 ± 0.18	4.39 ± 0.26	4.51 ± 0.27	0.024 (-0.101, 0.148)	0.71	0.069 (-0.007, 0.142)	0.07
SRL	FD	1.71 ± 0.01	1.68 ± 0.04	1.62 ± 0.06	-0.022 (-0.034, -0.011)	< 0.001	-0.041 (-0.055, -0.026)	< 0.001
	VD	0.43 ± 0.01	0.36 ± 0.05	0.29 ± 0.07	-0.054 (-0.071, -0.037)	< 0.001	-0.065 (-0.084, -0.046)	< 0.001
	SD	0.094 ± 0.004	0.08 ± 0.01	0.06 ± 0.02	-0.011 (-0.015, -0.006)	< 0.001	-0.015 (-0.019, -0.010)	< 0.001
	VDI	4.56 ± 0.22	4.49 ± 0.23	4.63 ± 0.21	-0.054 (-0.174, 0.067)	0.38	0.026 (-0.040, 0.091)	0.44
DRL	FD	1.72 ± 0.01	1.71 ± 0.03	1.69 ± 0.03	-0.005 (-0.015, 0.007)	0.43	-0.010 (-0.019, -0.002)	0.01
	VD	0.42 ± 0.01	0.40 ± 0.05	0.37 ± 0.06	-0.012 (-0.031, 0.006)	0.20	-0.022 (-0.037, -0.008)	0.003
	SD	0.098 ± 0.004	0.09 ± 0.01	0.09 ± 0.01	-0.003 (-0.007, 0.002)	0.29	-0.005 (-0.009, -0.002)	0.002
	VDI	4.31 ± 0.14	4.30 ± 0.16	4.35 ± 0.19	-0.007 (-0.090, 0.076)	0.87	0.008 (-0.047, 0.062)	0.78

BRVO = branch retinal venous occlusion; CRVO = central retinal venous occlusion; NS-RL = nonsegmented retina layer; SRL = superficial retina layer; DRL = deeper retina layer; FD = fractal dimension; VD = vessel density; SD = skeletal density; VDI = vessel diameter index; β = age and gender adjusted linear regression coefficient; CI = confidence interval

*P-values were estimated from nonparametric linear regressions.

Compared to the unaffected fellow eye, mean FD, VD, and SD were significantly lower in CRVO and BRVO eyes (p<0.001). Mean VDI was significantly higher for both groups (p≤0.03) as detailed in S3 and S4 Tables.

**Fig 3:**
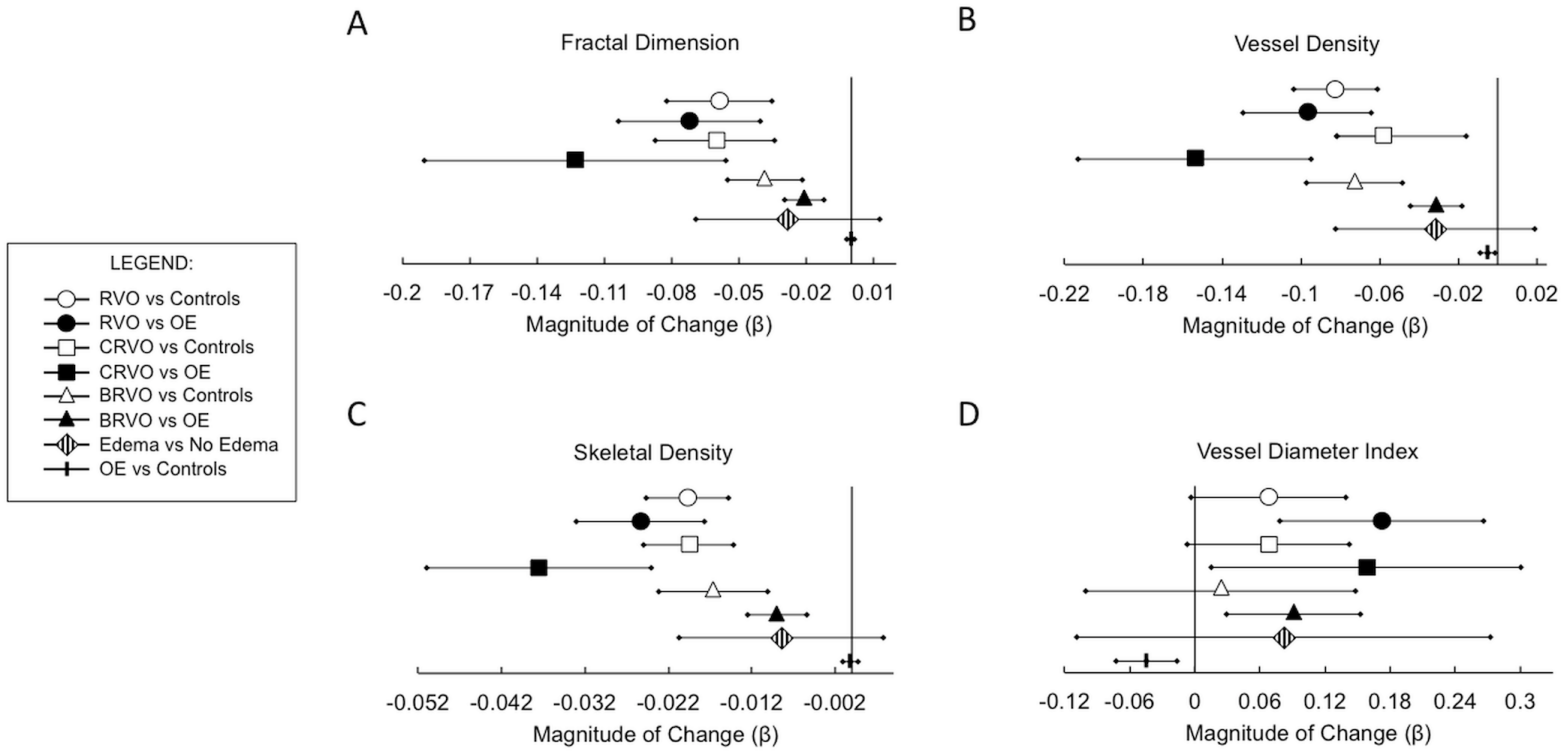
Magnitude and direction of change in quantitative OCTA parameters among all study subjects using nonsegmented layer analysis. Linear regression slope coefficient (β) indicating magnitude and direction of parameter difference, and corresponding confidence intervals. Abbreviations: RVO, retinal venous occlusion, OE, other (unaffected fellow) eye; CRVO, central retinal venous.

In the NS-RL analysis, as the extent of occlusion increased (control vs BRVO vs CRVO), mean FD, VD, and SD were progressively lower (p<0.001, Jonckheere-Terpstra test). No significant difference was observed between the VDI means of these two groups (p = 0.34). Please refer to Fig 3 for a summary of the nonsegmented β values that represent the magnitude and direction of parameter changes.

### Segmented analysis of retinal capillaries for all RVO

Thirty-two RVO eyes underwent segmented analysis of the SRL and DRL. The SRL of RVO eyes demonstrated significantly lower mean FD, VD, and SD (p<0.001) when compared to controls. VDI was unchanged (p = 0.46). Compared to the unaffected fellow eye, the mean FD, VD and SD were also significantly lower (p<0.001), and VDI significantly greater (p = 0.008; refer to S1 Table for details). The magnitude of the difference in mean FD, VD, and SD correlated with the extent of occlusion (control vs BRVO vs CRVO; p<0.001).

In the DRL, RVO eyes demonstrated significantly lower mean FD (p = 0.008), VD (p = 0.001), and SD (p = 0.001) compared to controls. VDI was unchanged (p = 0.44). When compared to the unaffected fellow eye, the mean FD, VD and SD were also significantly lower (p≤0.004), and mean VDI significantly higher (p = 0.005; S1 Table). In the DRL, the magnitude of the difference in mean VD and SD correlated with the extent of occlusion (p = 0.003 and p = 0.002, respectively).

### Segmented retina analysis of CRVO eyes

Compared to control eyes, the SRL of CRVO eyes demonstrated significantly lower mean values for FD, VD, and SD (p<0.001). No difference was found in VDI. (p = 0.44). The same trends were observed in the DRL, with similar significance, as detailed in Table 2.

Compared to the unaffected fellow eye, the SRL vasculature of CRVO eyes had significantly lower mean values for FD, VD and SD (p<0.001); mean VDI values were not significantly different (p = 0.07). In the DRL, mean FD, VD, and SD values for CRVO eyes were significantly lower compared to fellow eyes (p≤0.01), while VDI was significantly greater (p = 0.03; refer to S3 Table for details).

### Segmented retina analysis of BRVO eyes

Compared to control eyes, the SRL in BRVO eyes demonstrated lower mean FD, VD, and SD (p<0.001), and VDI was unchanged (p = 0.38). In contrast, none of the parameters were statistically different for BRVO eyes in the DRL (p≥0.20; see Table 2).

Compared to the unaffected fellow eye, the SRL in BRVO eyes demonstrated a significant lower mean FD, VD, and SD (p<0.001). VDI was not significantly different (p = 0.052). In the DRL, BRVO eyes demonstrated only a significant lower mean FD (p = 0.049) and SD (p = 0.03; refer to S4 Table for details).

### OCTA findings in CRVO eyes compared to BRVO eyes

CRVO eyes demonstrated lower mean FD, VD and SD in all layers compared to BRVO eyes (p<0.002 for NS-RL, p<0.001 for SRL, and p<0.03 for DRL). Similarly, CRVO eyes demonstrated a trend towards higher mean VDI in all layers, albeit insignificantly: p = 0.16 for NS-RL, p = 0.08 for SRL, and p = 0.34 for DRL. See S5 Table for details.

### OCTA findings in RVO eyes with macular edema

Twenty-two (64.7%) RVO eyes had macular edema on the date of imaging. NS-RL analysis revealed no significant difference between RVO eyes with and without macular edema. In the SRL, eyes with edema demonstrated a significantly lower mean FD, VD and SD (p≤0.03), while VDI remained unchanged (p = 0.51) compared to eyes without edema. In the DRL, no differences were observed (p≥0.20; refer to S6 Table).

### Comparing OCTA findings in fellow eyes to control eyes

The fellow, unaffected eye of subjects with RVO was compared to control eyes to determine if there were sub-clinical changes that may correlate with RVO status. In the NS-RL, the fellow eye demonstrated significantly lower mean VD (p = 0.009) and VDI (p = 0.002) compared to control eyes (Fig 2A). Similarly in the SRL, significantly lower mean VD (p = 0.006) and VDI (p = 0.001) were observed. In the DRL, the fellow eyes demonstrated significantly higher mean FD (p = 0.006) and SD (p = 0.03), and lower VDI (p = 0.008; S7 Table).

The Jonckheere-Terpstra test demonstrated statistically significant parameter trends as RVO status changed (control eyes vs fellow eyes vs RVO eyes). In the NS-RL and SRL, three parameters (FD, VD and SD) progressively and significantly decreased (p<0.001), while VDI was not different (p≥0.54). In the DRL, no significant trends were observed (p≥0.17).

## Discussion

We have demonstrated that OCTA-based metrics of capillary morphology and density have a significant association with clinical severity of disease among subjects with RVO. Specifically, capillary morphology (FD, VDI) and density (VD, SD) are significantly different in RVO eyes compared to unaffected fellow eyes. Compared to control eyes, all parameters except VDI are significantly different in RVO eyes. These findings are most reliably noted when analyzing the capillaries from the nonsegmented retina as well as the SRL. Findings in the DRL are less robust but show the same trends. These quantitative, layer-specific observations are consistent with qualitative OCTA observations, which include impaired capillary perfusion (decreased capillary density) and increased vessel caliber.[4, 8, 17] Furthermore, we demonstrated that RVO eyes with macular edema show greater vascular change compared to those without edema in the SRL. A similar trend was observed in the NS-RL and DRL, albeit insignificantly.

Impaired capillary perfusion, typically assessed by IVFA, is a hallmark of RVO that is known to change over time.[18, 19] Traditional IVFAs are not repeated in the clinical setting frequently and subsequent ischemic changes are often indirectly inferred by the presence of macular edema, neovascularization, or changes in vision. When IVFAs are repeated in a serial fashion, the changes that are noted are characterized in a subjective or qualitative manner. By contrast, OCTA provides a rapid and noninvasive method of following the severity of macular ischemia in RVO subjects quantitatively as well as qualitatively. We have demonstrated the feasibility of OCTA for objective quantitation of RVO severity. Compared to BRVO subjects, CRVO subjects demonstrated a greater difference in the magnitude (β) of the OCTA parameter values (Table 2). This is noteworthy since the parameters are derived from a 3x3mm macular scan. This suggests that quantitative OCTA measurements of the central macula are a potentially useful biomarker of disease severity in RVO.

Vascular engorgement, vessel tortuosity, and development of shunt vessels are findings that are commonly observed in RVO. These are observed clinically at the macrovascular level. There are no quantitative metrics to assess these changes *in vivo* at the capillary level. We show that RVO eyes demonstrated greater capillary caliber (VDI) compared to the fellow eye that serves as an internal control. Interestingly, the capillary caliber of the unaffected fellow eye was significantly lower compared to healthy, control eyes in our study. Since hypertension is a major risk factor for RVO—and given the predominance of hypertension in our RVO cohort—the lower capillary caliber in the fellow eye of subjects with RVO may represent a manifestation of chronic hypertension and its attendant vascular attenuation. While our results are based on a small sample size and must be interpreted with caution, these results also suggest that changes in capillary caliber may be an important biomarker of disease and require further attention in clinical studies. Interestingly, Pinhas et al. studied the uninvolved, fellow eye of subjects with nonischemic CRVO using adaptive optics scanning laser ophthalmoscopy, and noted areas of abnormal capillary perfusion in 90% of fellow eyes.[20] Furthermore, the microvascular density of the fellow eye was greater than the density of the RVO eye, but lower compared to healthy controls. They did not report on vascular caliber, but our findings are consistent with their general observations. In addition, OCTA of subjects with RVO before and after anti-vascular endothelial growth factor treatment shows changes in capillary density as well significantly lower capillary density than controls.[21]

CRVO and BRVO eyes independently demonstrated a statistically significant lower FD, VD and SD in the SRL when compared to controls and to the unaffected fellow eyes. In the DRL, only CRVO eyes demonstrated these trends. When compared to the other eye, vessel caliber (VDI) was significantly greater for CRVO eyes in the DRL. The significantly greater vessel caliber observed in CRVO eyes in the DRL compared to the SRL may suggest that the deep vascular plexus vessels are more altered than the SRL in subjects with RVO, as suggested by Coscas et al.[17] One possible explanation is that the deep capillary plexus is a relative watershed zone between the retinal vascular and choroidal circulations, and may therefore be prone to ischemic alterations.

Nevertheless, the discrepancy between the SRL and DRL findings must be interpreted with caution. OCTA images, and therefore binarized and skeletonized images, are subject to a number of artifacts (e.g., vitreous opacities, cataracts, or other media opacities) including projection artifacts from inner retinal vessels onto outer retinal layers, which could impact results. Our current analyses suggest that OCTA-based capillary density measurements are consistent with histology based assessments.[6] Therefore, we have confidence that the findings in the SRL are representative of pathologic changes in our RVO cohort. Changes in the DRL are consistent with SRL findings in most of our comparisons; however, the magnitude of change in the DRL may be blunted by projection artifacts. Interestingly, the presence and magnitude of DRL changes is most notable in the most severe cases of RVO, which suggests that overlying capillaries in mild RVO cases may blunt DRL changes. Zhang et al. recently reported a novel algorithm to minimize projection artifacts in the deeper retinal layers.[22] Future studies with improved segmentation methods that incorporate this artifact correction algorithm may help to address DRL changes more carefully.

Our study was limited by several considerations. First, our study has a relatively small sample size and is retrospective in nature. Inclusion of subjects with very poor visual acuity was not possible due to their inability to fixate during image acquisition. Second, we studied RVO eyes that received treatments and those that were treatment naïve in a cross-sectional design. As it would be unethical to withhold treatment from RVO subjects with macular edema, it is unlikely that a systematic comparative study of treated and untreated eyes will be possible. In order to control for any systematic differences between these groups we attempted to perform a sub-group analysis of treated and treatment naïve eyes and we did not find any significant differences in OCTA parameters between these sub-groups (data not shown). Third, our age and gender-matched controls included subjects with comorbidities such as systemic hypertension and diabetes, which could have confounded our results. Due to the very high prevalence of these systemic conditions in the age-matched control population it is not feasible to recruit a completely healthy control group in a single center study of this size. However, any potential confounding effect of these comorbidities in our real-world control group would likely serve to decrease the magnitude of our findings rather than completely invalidate our results. With a completely healthy control group, one would reasonably expect larger and more significant differences in the OCTA parameters than we described with our current cohort. Lastly, our analysis was restricted to the vasculature within the 3x3mm fovea-centered macula region. Areas beyond this 3x3mm region were not evaluated, which could have limited our ability to detect mild microvascular changes.

In conclusion, OCTA provides noninvasive, quantitative analysis of RVO-associated microvascular changes. The parameters we describe provide an objective tool for monitoring microvascular disease severity and may be useful in assessing changes over time and with treatment. Quantitative OCTA analysis of retinal capillaries in the central 3x3mm area of the macula demonstrates that there are significant differences in capillary density and morphology between subjects with BRVO, CRVO and control subjects. The unaffected fellow eyes of subjects with RVO also demonstrate significant retinal vascular changes compared to healthy control eyes and the fellow RVO eye. Quantitative OCTA analysis of capillary density and morphology may represent a useful measure for clinical evaluation, response to treatment, and overall disease management.

## Supporting information

S1 Fig Representative OCTA images of the retinal vascular layers of interest. B-scan images highlighting with purple dotted line the three retinal vascular layers of interest: Nonsegmented whole retina, NS-RL (A), superficial retina layer, SRL (E), and deeper retina layer, DRL (I). Corresponding angiograms (B,F,J), binarized images (C,G,K), and skeletonized images (D,H,L).
(TIFF)

S1 Table Comparison of quantitative OCTA parameters between RVO eyes and fellow unaffected eyes or control eyes. RVO = retinal venous occlusion; OE = other (unaffected fellow) eye of subjects with RVO; NS-RL = nonsegmented retina layer; SRL = superficial retina layer; DRL = deeper retina layer; FD = fractal dimension; VD = vessel density; SD = skeletal density; VDI = vessel diameter index; β = unranked linear regression slope coefficient; CI = confidence interval.
(DOCX)

S2 Table Point-biserial correlation analysis for RVO eyes and OCTA metrics. *r*_pb_ = point-biserial correlation coefficient; RVO = retinal venous occlusion; BRVO = branch retinal venous occlusion; CRVO = central retinal venous occlusion; NS-RL = nonsegmented retina layer; SRL = superficial retina layer; DRL = deeper retina layer; FD = fractal dimension; VD = vessel density; SD = skeletal density; VDI = vessel diameter index.
(DOCX)

S3 Table Comparison of quantitative OCTA parameters between CRVO eyes and fellow unaffected eyes. * Indicates there was a statistically significant difference (p<0.05) between CRVO eyes and the fellow eye. CRVO = central retinal venous occlusion; OE = other (unaffected fellow) eye; NS-RL = nonsegmented retina layer; SRL = superficial retina layer; DRL = deeper retina layer; FD = fractal dimension; VD = vessel density; SD = skeletal density; VDI = vessel diameter index; β = unranked linear regression slope coefficient; CI = confidence interval.
(DOCX)

S4 Table Comparison of quantitative OCTA parameters between BRVO eyes and fellow unaffected eyes. * Indicates there was a statistically significant difference (p<0.05) between BRVO eyes and the fellow eye. BRVO = branch retinal venous occlusion; OE = other (unaffected fellow) eye; NS-RL = nonsegmented retina layer; SRL = superficial retina layer; DRL = deeper retina layer; FD = fractal dimension; VD = vessel density; SD = skeletal density; VDI = vessel diameter index; β = unranked linear regression slope coefficient; CI = confidence interval.
(DOCX)

S5 Table Comparison of quantitative OCTA parameters between CRVO and BRVO eyes. * Indicates there was a statistically significant difference (p<0.05) between CRVO and BRVO eyes. BRVO = branch retinal venous occlusion; CRVO = central retinal venous occlusion; NS-RL = nonsegmented retina layer; SRL = superficial retina layer; DRL = deeper retina layer; FD = fractal dimension; VD = vessel density; SD = skeletal density; VDI = vessel diameter index; β = unranked linear regression slope coefficient; CI = confidence interval.
(DOCX)

S6 Table Comparison of quantitative OCTA parameters between RVO eyes with and without macular edema. * Indicates there was a statistically significant difference (p<0.05) between RVO eyes with and without macular edema. RVO = retinal venous occlusion; NS-RL = nonsegmented retina layer; SRL = superficial retina layer; DRL = deeper retina layer; FD = fractal dimension; VD = vessel density; SD = skeletal density; VDI = vessel diameter index; β = unranked linear regression slope coefficient; CI = confidence interval.
(DOCX)

S7 Table Comparison of quantitative OCTA parameters between fellow unaffected eyes and control eyes. * Indicates there was a statistically significant difference (p<0.05) between control eyes and the fellow unaffected eye of subjects with RVO. OE = other (unaffected fellow) eye of subjects with RVO; NS-RL = nonsegmented retina layer; SRL = superficial retina layer; DRL = deeper retina layer; FD = fractal dimension; VD = vessel density; SD = skeletal density; VDI = vessel diameter index; β = unranked linear regression slope coefficient; CI = confidence interval.
(DOCX)
