# Supplementary figures and images for: Quantitative microvascular analysis of retinal venous occlusions by spectral domain optical coherence tomography angiography
Source: PLoS One. 2017 Apr 24;12(4):e0176404. doi: 10.1371/journal.pone.0176404 (PMC5402954; doi:10.1371/journal.pone.0176404)

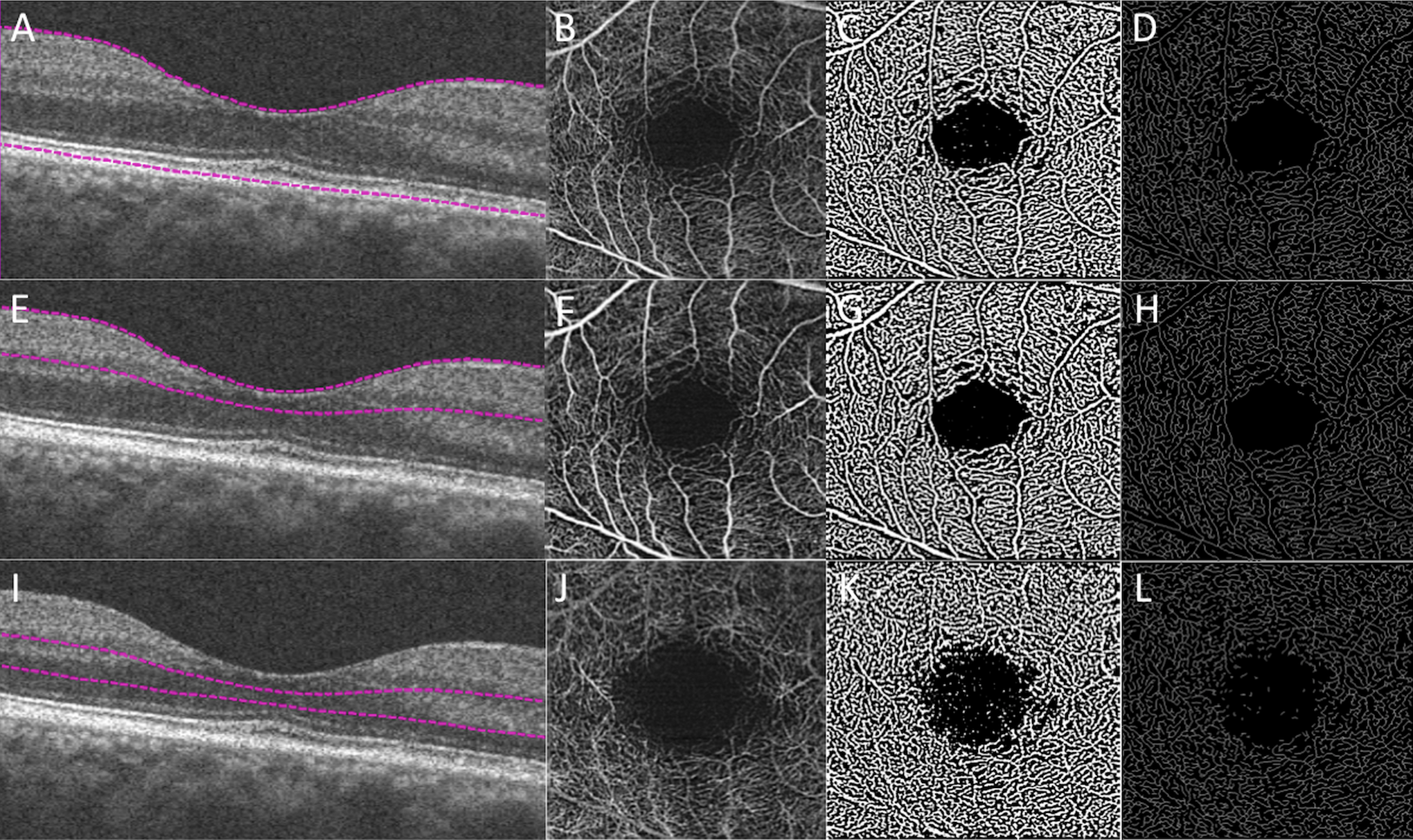

Supplement: S1 Fig — B-scan images highlighting with purple dotted line the three retinal vascular layers of interest: Nonsegmented whole retina, NS-RL (A), superficial retina layer, SRL (E), and deeper retina layer, DRL (I). Corresponding angiograms (B,F,J), binarized images (C,G,K), and skeletonized images (D,H,L). (TIFF) [file pone.0176404.s001.tiff]
