# Supplementary material for: Quantitative microvascular analysis of retinal venous occlusions by spectral domain optical coherence tomography angiography
Source: PLoS One. 2017 Apr 24;12(4):e0176404. doi: 10.1371/journal.pone.0176404 (PMC5402954; doi:10.1371/journal.pone.0176404)
Supplement: S1 Table — RVO = retinal venous occlusion; OE = other (unaffected fellow) eye of subjects with RVO; NS-RL = nonsegmented retina layer; SRL = superficial retina layer; DRL = deeper retina layer; FD = fractal dimension; VD = vessel density; SD = skeletal density; VDI = vessel diameter index; β = unranked linear regression slope coefficient; CI = confidence interval. (DOCX) [file pone.0176404.s002.docx]

|  |  | **Controls** | **OE** | **RVO** | **RVO vs Other Eye** | | **RVO vs Controls** | |
| --- | --- | --- | --- | --- | --- | --- | --- | --- |
|  |  | **Mean ± SD** | **Mean ± SD** | **Mean ± SD** | **β (CI)** | **p-value** | **β (CI)** | **p-value** |
| NS-RL | FD | 1.72 ± 0.01 | 1.71 ± 0.01 | 1.64 ± 0.08 | -0.072 (-0.104, -0.041) | < 0.001 | -0.059 (-0.082, -0.035) | < 0.001 |
|  | VD | 0.43 ± 0.01 | 0.41 ± 0.03 | 0.32 ± 0.09 | -0.097 (-0.130, -0.064) | < 0.001 | -0.082 (-0.104, -0.061) | < 0.001 |
|  | SD | 0.099 ± 0.004 | 0.10 ± 0.01 | 0.07 ± 0.02 | -0.025 (-0.033, -0.018) | < 0.001 | -0.020 (-0.025, -0.015) | < 0.001 |
|  | VDI | 4.37 ± 0.18 | 4.24 ± 0.15 | 4.41 ± 0.27 | 0.173 (0.078, 0.266) | < 0.001 | 0.068 (-0.004, 0.139) | 0.06 |
| SRL | FD | 1.71 ± 0.01 | 1.71 ± 0.01 | 1.66 ± 0.05 | -0.042 (-0.061, -0.024) | < 0.001 | -0.039 (-0.053, -0.026) | < 0.001 |
|  | VD | 0.43 ± 0.01 | 0.41 ± 0.03 | 0.34 ± 0.07 | -0.066 (-0.091, -0.041) | < 0.001 | -0.065 (-0.082, -0.048) | < 0.001 |
|  | SD | 0.094 ± 0.004 | 0.09 ± 0.01 | 0.08 ± 0.02 | -0.016 (-0.022, -0.010) | < 0.001 | -0.015 (-0.019, -0.011) | < 0.001 |
|  | VDI | 4.56 ± 0.22 | 4.41 ± 0.12 | 4.51 ± 0.18 | 0.095 (0.025, 0.165) | 0.008 | 0.025 (-0.041, 0.090) | 0.46 |
| DRL | FD | 1.72 ± 0.01 | 1.72 ± 0.01 | 1.70 ± 0.03 | -0.020 (-0.034, -0.007) | 0.003 | -0.011 (-0.020, -0.003) | 0.008 |
|  | VD | 0.42 ± 0.01 | 0.42 ± 0.01 | 0.39 ± 0.06 | -0.033 (-0.056, -0.011) | 0.004 | -0.023 (-0.037, -0.009) | 0.001 |
|  | SD | 0.098 ± 0.004 | 0.100 ± 0.005 | 0.09 ± 0.01 | -0.009 (-0.015, -0.004) | 0.001 | -0.006 (-0.009, -0.002) | 0.001 |
|  | VDI | 4.31 ± 0.14 | 4.23 ± 0.09 | 4.31 ± 0.14 | 0.078 (0.023, 0.132) | 0.005 | 0.017 (-0.036, 0.070) | 0.54 |
