# Supplementary material for: Quantitative microvascular analysis of retinal venous occlusions by spectral domain optical coherence tomography angiography
Source: PLoS One. 2017 Apr 24;12(4):e0176404. doi: 10.1371/journal.pone.0176404 (PMC5402954; doi:10.1371/journal.pone.0176404)
Supplement: S2 Table — rpb = point-biserial correlation coefficient; RVO = retinal venous occlusion; BRVO = branch retinal venous occlusion; CRVO = central retinal venous occlusion; NS-RL = nonsegmented retina layer; SRL = superficial retina layer; DRL = deeper retina layer; FD = fractal dimension; VD = vessel density; SD = skeletal density; VDI = vessel diameter index. (DOCX) [file pone.0176404.s003.docx]

|  |  | **BRVO vs Controls**  ***r*_pb_ (p-value)** | **CRVO vs Controls**  ***r*_pb_ (p-value)** |
| --- | --- | --- | --- |
| NS-RL | FD | -0.57436 (<0.001) | -0.78739 (<0.001) |
|  | VD | -0.79465 (<0.001) | -0.7901 (<0.001) |
|  | SD | -0.58482 (<0.001) | -0.78153 (<0.001) |
|  | VDI | 0.01130 (0.94) | 0.18383 (0.26) |
| SRL | FD | -0.49083 (<0.001) | -0.74201 (<0.001) |
|  | VD | -0.79442 (<0.001) | -0.75997 (<0.001) |
|  | SD | -0.55008 (<0.001) | -0.73014 (<0.001) |
|  | VDI | -0.11489 (0.46) | 0.114777 (0.38) |
| DRL | FD | 0.04673 (0.76) | -0.45637 (0.004) |
|  | VD | -0.03910 (0.80) | -0.48950 (0.002) |
|  | SD | -0.00169 (0.99) | -0.46763 (0.003) |
|  | VDI | -0.05374 (0.73) | 0.08114 (0.63) |
