# Supplementary material for: Quantitative microvascular analysis of retinal venous occlusions by spectral domain optical coherence tomography angiography
Source: PLoS One. 2017 Apr 24;12(4):e0176404. doi: 10.1371/journal.pone.0176404 (PMC5402954; doi:10.1371/journal.pone.0176404)
Supplement: S3 Table — * Indicates there was a statistically significant difference (p<0.05) between CRVO eyes and the fellow eye. CRVO = central retinal venous occlusion; OE = other (unaffected fellow) eye; NS-RL = nonsegmented retina layer; SRL = superficial retina layer; DRL = deeper retina layer; FD = fractal dimension; VD = vessel density; SD = skeletal density; VDI = vessel diameter index; β = unranked linear regression slope coefficient; CI = confidence interval. (DOCX) [file pone.0176404.s004.docx]

|  |  | **OE**  **Mean ± SD** | **CRVO** | **CRVO vs Other Eye** | |
| --- | --- | --- | --- | --- | --- |
|  |  |  | **Mean ± SD** | **β (CI)** | **p-value** |
| NS-RL | FD * | 1.71 ± 0.01 | 1.59 ± 0.11 | -0.123 (-0.190, -0.056) | < 0.001 |
|  | VD | 0.41 ± 0.03 | 0.26 ± 0.10 | -0.154 (-0.213, -0.095) | < 0.001 |
|  | SD * | 0.10 ± 0.01 | 0.06 ± 0.02 | -0.038 (-0.051, -0.024) | < 0.001 |
|  | VDI * | 4.30 ± 0.18 | 4.46 ± 0.29 | 0.158 (0.016, 0.301) | 0.03 |
| SRL | FD * | 1.70 ± 0.02 | 1.63 ± 0.06 | -0.074 (-0.111, -0.037) | < 0.001 |
|  | VD * | 0.40 ± 0.03 | 0.29 ± 0.08 | -0.107 (-0.154, -0.061) | < 0.001 |
|  | SD * | 0.09 ± 0.01 | 0.06 ± 0.02 | -0.026 (-0.037, -0.015) | < 0.001 |
|  | VDI | 4.44 ± 0.14 | 4.55 ± 0.13 | 0.106 (-0.005, 0.216) | 0.07 |
| DRL | FD * | 1.72 ± 0.01 | 1.69 ± 0.04 | -0.035 (-0.062, -0.007) | 0.01 |
|  | VD * | 0.42 ± 0.02 | 0.36 ± 0.07 | -0.061 (-0.106, -0.017) | 0.007 |
|  | SD * | 0.10 ± 0.01 | 0.08 ± 0.02 | -0.016 (-0.028, -0.005) | 0.006 |
|  | VDI * | 4.25 ± 0.10 | 4.35 ± 0.11 | 0.095 (0.008, 0.182) | 0.03 |
