# Supplementary material for: Quantitative microvascular analysis of retinal venous occlusions by spectral domain optical coherence tomography angiography
Source: PLoS One. 2017 Apr 24;12(4):e0176404. doi: 10.1371/journal.pone.0176404 (PMC5402954; doi:10.1371/journal.pone.0176404)
Supplement: S4 Table — * Indicates there was a statistically significant difference (p<0.05) between BRVO eyes and the fellow eye. BRVO = branch retinal venous occlusion; OE = other (unaffected fellow) eye; NS-RL = nonsegmented retina layer; SRL = superficial retina layer; DRL = deeper retina layer; FD = fractal dimension; VD = vessel density; SD = skeletal density; VDI = vessel diameter index; β = unranked linear regression slope coefficient; CI = confidence interval. (DOCX) [file pone.0176404.s005.docx]

|  |  | **OE**  **Mean ± SD** | **BRVO**  **Mean ± SD** | **BRVO vs Other Eye** | |
| --- | --- | --- | --- | --- | --- |
|  |  |  |  | **β (CI)** | **p-value** |
| NS-RL | FD * | 1.72 ± 0.01 | 1.67 ± 0.04 | -0.021 (-0.030, -0.012) | < 0.001 |
|  | VD * | 0.42 ± 0.03 | 0.35 ± 0.05 | -0.031 (-0.045, -0.018) | < 0.001 |
|  | SD * | 0.10 ± 0.01 | 0.08 ± 0.01 | -0.009 (-0.013, -0.005) | < 0.001 |
|  | VDI * | 4.20 ± 0.12 | 4.39 ± 0.27 | 0.091 (0.029, 0.153) | 0.004 |
| SRL | FD * | 1.71 ± 0.01 | 1.67 ± 0.04 | -0.012 (-0.018, -0.006) | < 0.001 |
|  | VD * | 0.41 ± 0.02 | 0.36 ± 0.06 | -0.021 (-0.032, -0.011) | < 0.001 |
|  | SD * | 0.094 ± 0.004 | 0.08 ± 0.01 | -0.006 (-0.008, -0.003) | < 0.001 |
|  | VDI | 4.40 ± 0.11 | 4.47 ± 0.22 | 0.045 (-0.0004, 0.090) | 0.052 |
| DRL | FD * | 1.72 ± 0.01 | 1.70 ± 0.04 | -0.006 (-0.012, 0.0000) | 0.049 |
|  | VD | 0.42 ± 0.01 | 0.40 ± 0.05 | -0.009 (-0.019, 0.001) | 0.09 |
|  | SD * | 0.100 ± 0.004 | 0.09 ± 0.01 | -0.003 (-0.005, -0.0003) | 0.03 |
|  | VDI | 4.22 ± 0.09 | 4.29 ± 0.16 | 0.034 (-0.001, 0.009) | 0.06 |
