# Supplementary material for: Quantitative microvascular analysis of retinal venous occlusions by spectral domain optical coherence tomography angiography
Source: PLoS One. 2017 Apr 24;12(4):e0176404. doi: 10.1371/journal.pone.0176404 (PMC5402954; doi:10.1371/journal.pone.0176404)
Supplement: S5 Table — * Indicates there was a statistically significant difference (p<0.05) between CRVO and BRVO eyes. BRVO = branch retinal venous occlusion; CRVO = central retinal venous occlusion; NS-RL = nonsegmented retina layer; SRL = superficial retina layer; DRL = deeper retina layer; FD = fractal dimension; VD = vessel density; SD = skeletal density; VDI = vessel diameter index; β = unranked linear regression slope coefficient; CI = confidence interval. (DOCX) [file pone.0176404.s006.docx]

|  |  | **BRVO**  **Mean ± SD** | **CRVO** | **CRVO vs BRVO** | |
| --- | --- | --- | --- | --- | --- |
|  |  |  | **Mean ± SD** | **β (CI)** | **p-value** |
| NS-RL | FD * | 1.68 ± 0.04 | 1.59 ± 0.10 | -0.0850 (-0.1387, -0.0313) | 0.002 |
|  | VD * | 0.36 ± 0.06 | 0.26 ± 0.09 | -0.0959 (-0.1486, -0.0433) | < 0.001 |
|  | SD * | 0.08 ± 0.02 | 0.06 ± 0.02 | -0.0241 (-0.0368, -0.0114) | < 0.001 |
|  | VDI | 4.39 ± 0.26 | 4.51 ± 0.27 | 0.1327 (-0.0509, 0.3164) | 0.16 |
| SRL | FD * | 1.68 ± 0.04 | 1.62 ± 0.06 | -0.0627 (-0.0940, -0.0314) | < 0.001 |
|  | VD * | 0.36 ± 0.05 | 0.29 ± 0.07 | -0.0815 (-0.1225, -0.0405) | < 0.001 |
|  | SD * | 0.08 ± 0.01 | 0.06 ± 0.02 | -0.0203 (-0.0301, -0.0105) | < 0.001 |
|  | VDI | 4.49 ± 0.23 | 4.63 ± 0.21 | 0.1352 (-0.0138, 0.2841) | 0.08 |
| DRL | FD * | 1.71 ± 0.03 | 1.69 ± 0.03 | -0.0220 (-0.0416, -0.0025) | 0.03 |
|  | VD * | 0.40 ± 0.05 | 0.37 ± 0.06 | -0.0401 (-0.0724, -0.0077) | 0.02 |
|  | SD * | 0.09 ± 0.01 | 0.09 ± 0.01 | -0.0106 (-0.0187, -0.0024) | 0.01 |
|  | VDI | 4.30 ± 0.16 | 4.35 ± 0.19 | 0.0601 (-0.0637, 0.1839) | 0.34 |
