# Supplementary material for: Quantitative microvascular analysis of retinal venous occlusions by spectral domain optical coherence tomography angiography
Source: PLoS One. 2017 Apr 24;12(4):e0176404. doi: 10.1371/journal.pone.0176404 (PMC5402954; doi:10.1371/journal.pone.0176404)
Supplement: S6 Table — * Indicates there was a statistically significant difference (p<0.05) between RVO eyes with and without macular edema. RVO = retinal venous occlusion; NS-RL = nonsegmented retina layer; SRL = superficial retina layer; DRL = deeper retina layer; FD = fractal dimension; VD = vessel density; SD = skeletal density; VDI = vessel diameter index; β = unranked linear regression slope coefficient; CI = confidence interval. (DOCX) [file pone.0176404.s007.docx]

|  |  | **No Edema**  **Mean ± SD** | **Edema** | **Edema vs No Edema** | |
| --- | --- | --- | --- | --- | --- |
|  |  |  | **Mean ± SD** | **β (CI)** | **p-value** |
| NS-RL | FD | 1.66 ± 0.06 | 1.63 ± 0.09 | -0.029 (-0.070, 0.013) | 0.17 |
|  | VD | 0.34 ± 0.08 | 0.31 ± 0.09 | -0.032 (-0.082, 0.019) | 0.22 |
|  | SD | 0.08 ± 0.02 | 0.07 ± 0.02 | -0.008 (-0.021, 0.004) | 0.18 |
|  | VDI | 4.40 ± 0.32 | 4.47 ± 0.24 | 0.082 (-0.109, 0.273) | 0.40 |
| SRL | FD * | 1.68 ± 0.05 | 1.65 ± 0.05 | -0.029 (-0.055, -0.004) | 0.03 |
|  | VD * | 0.36 ± 0.06 | 0.32 ± 0.07 | -0.039 (-0.075, -0.003) | 0.03 |
|  | SD * | 0.08 ± 0.02 | 0.07 ± 0.02 | -0.010 (-0.018, -0.001) | 0.02 |
|  | VDI | 4.53 ± 0.29 | 4.58 ± 0.16 | 0.058 (-0.116, 0.232) | 0.51 |
| DRL | FD | 1.71 ± 0.03 | 1.70 ± 0.02 | -0.010 (-0.029, 0.009) | 0.31 |
|  | VD | 0.41 ± 0.05 | 0.39 ± 0.05 | -0.019 (-0.050, 0.012) | 0.23 |
|  | SD | 0.10 ± 0.01 | 0.09 ± 0.01 | -0.005 (-0.013, 0.003) | 0.20 |
|  | VDI | 4.31 ± 0.17 | 4.33 ± 0.17 | 0.023 (-0.090, 0.137) | 0.69 |
