# Supplementary material for: Quantitative microvascular analysis of retinal venous occlusions by spectral domain optical coherence tomography angiography
Source: PLoS One. 2017 Apr 24;12(4):e0176404. doi: 10.1371/journal.pone.0176404 (PMC5402954; doi:10.1371/journal.pone.0176404)
Supplement: S7 Table — * Indicates there was a statistically significant difference (p<0.05) between control eyes and the fellow unaffected eye of subjects with RVO. OE = other (unaffected fellow) eye of subjects with RVO; NS-RL = nonsegmented retina layer; SRL = superficial retina layer; DRL = deeper retina layer; FD = fractal dimension; VD = vessel density; SD = skeletal density; VDI = vessel diameter index; β = unranked linear regression slope coefficient; CI = confidence interval. (DOCX) [file pone.0176404.s008.docx]

|  |  | **Controls**  **Mean ± SD** | **OE** | **OE vs Control** | |
| --- | --- | --- | --- | --- | --- |
|  |  |  | **Mean ± SD** | **β (CI)** | **p-value** |
| NS-RL | FD | 1.72 ± 0.01 | 1.71 ± 0.01 | -0.0002 (-0.0020, 0.0016) | 0.83 |
|  | VD * | 0.43 ± 0.01 | 0.41 ± 0.03 | -0.005 (-0.009, -0.001) | 0.009 |
|  | SD | 0.099 ± 0.004 | 0.10 ± 0.01 | -0.0002 (-0.0011, 0.0008) | 0.70 |
|  | VDI * | 4.37 ± 0.18 | 4.24 ± 0.15 | -0.045 (-0.073, -0.016) | 0.002 |
| SRL | FD | 1.71 ± 0.01 | 1.71 ± 0.01 | -0.0004 (-0.0013, 0.0020) | 0.66 |
|  | VD * | 0.43 ± 0.01 | 0.41 ± 0.03 | -0.005 (-0.008, -0.001) | 0.006 |
|  | SD | 0.094 ± 0.004 | 0.09 ± 0.01 | -0.0000 (-0.0009, 0.0008) | 0.95 |
|  | VDI * | 4.56 ± 0.22 | 4.41 ± 0.12 | -0.052 (-0.083, -0.021) | 0.001 |
| DRL | FD * | 1.722 ± 0.008 | 1.717 ± 0.009 | 0.002 (0.001, 0.004) | 0.006 |
|  | VD | 0.420 ± 0.004 | 0.42 ± 0.01 | -0.001 (-0.002, 0.003) | 0.46 |
|  | SD * | 0.098 ± 0.004 | 0.100 ± 0.005 | 0.001 (0.0001, 0.0016) | 0.03 |
|  | VDI * | 4.31 ± 0.03 | 4.23 ± 0.09 | -0.027 (-0.047, -0.007) | 0.008 |
